# Laparoscopic Radiofrequency Ablation for Large Subcapsular Hepatic Hemangiomas: Technical and Clinical Outcomes

**DOI:** 10.1371/journal.pone.0149755

**Published:** 2016-02-22

**Authors:** Jun Gao, Jian-Song Ji, Xue-Mei Ding, Shan Ke, Zong-Hai Xin, Chun-Min Ning, Shi-Gang Guo, Xiao-Long Li, Yong-Hong Dong, Wen-Bing Sun

**Affiliations:** 1 Department of Hepatobiliary Surgery, Beijing Chao-yang Hospital Affiliated with Capital Medical University, No. 5 Jingyuan Street, Beijing 100043, China; 2 Department of Radiology, Lishui Central Hospital, The Fifth Affiliated Hospital of Wenzhou Medical College, Zhejiang, 32300, China; 3 Department of General Surgery, Zhanhua People's Hospital, Shandong 256800, China; 4 Department of General Surgery, Chaoyang Central Hospital, Liaoning 122000, China; 5 Department of General Surgery, Affiliated Hospital of Chifeng University, Neimenggu 024000, China; 6 Department of General Surgery, Shanxi Provincial People's Hospital, Shanxi 032200, China; The University of Hong Kong, HONG KONG

## Abstract

**Objectives:**

The aim of this study was to evaluate the technical and clinical outcomes of using laparoscopic radiofrequency (RF) ablation for treating large subcapsular hepatic hemangiomas.

**Methods:**

We retrospectively reviewed our sequential experience of treating 124 large subcapsular hepatic hemangiomas in 121 patients with laparoscopic RF ablation.

**Results:**

The mean diameter of the 124 hemangiomas was 9.1 ± 3.2 cm (5.0–16.0 cm). RF ablation was performed successfully in all patients. There were 55 complications related to the ablation in 26 patients, including 5 of 69 (7.3%) patients with hemangioma <10 cm and 21 of 52 (40.4%) patients with hemangiomas ≥10 cm (*P* < 0.001). No injuries to abdominal viscera occurred in all the 121 patients. According to the Dindo–Clavien classification, all the complications were minor in 26 patients (Grade I). Out of 124 hepatic hemangiomas, 118 (95.2%) were completely ablated, including 70 of 72 (97.2%) lesions < 10 cm and 48 of 52 (92.3%) lesions ≥ 10 cm (*P* = 0.236).

**Conclusion:**

Laparoscopic RF ablation therapy is a safe, feasible and effective procedure for large subcapsular hepatic hemangiomas, even in the hepatic hemangiomas ≥ 10 cm. Its use avoids thermal injury to the abdominal viscera.

## Introduction

Hepatic hemangiomas are the most common benign tumors occurring in human livers, with an incidence rate of 0.7% to 7% among general population, and are frequently diagnosed incidentally by abdominal imaging which is usually performed for other indications [1]. The majority of hemangiomas are small asymptomatic lesions that are managed by non-operative measures. Many studies have shown that small and asymptomatic hemangiomas can be left on a periodical schedule of imaging follow-up to check the status of the tumor mass without any intervention. Treatment is only needed for the tumor which is continuously growing and presents abdominal symptoms or is vulnerable to rupture [2–3].

Traditionally, surgical resection is the standard of care for enlarging symptomatic hepatic hemangiomas [4]. Although surgical resection can be used to completely remove the tumor, surgical procedure requires a large abdominal incision and is associated with relatively long hospitalization and high risk of morbidity [4]. In recent years, the rapid development of laparoscopic resection has provided great opportunities for treating hepatic hemangiomas in a minimally invasive fashion [5–7]. However, great technical challenges exist while using laparoscopic liver surgery for treating hepatic tumors and this technique is usually appropriate for treating patients with lesions located in the lower segments of livers [5–7]. Other minimally invasive procedures such as transarterial embolization or radiation therapy, have been attempted for treating symptomatic hepatic hemangiomas, but these technique are not curative treatments [8–9].

Radiofrequency (RF) ablation is an effective and minimally invasive treatment technique for managing hepatic hemangiomas with a dimension greater than 5 cm [10–13]. With the increasing experience on treating hepatic hemangiomas with RF ablation and the rapid improvement of RF equipment, this liver tumor treatment technique has also been performed successfully for tumor larger than 10 cm in diameter [14, 15].

The RF procedure can be performed via a percutaneous, laparoscopic approach or a laparotomic approach. Each approach has its own advantages and disadvantages [16]. The percutaneous approach can be performed under general or local anesthesia as an outpatient procedure. However, percutaneous RF ablation has great limitation for treating subcapsular tumors or lesions with a location in a proximity to pivotal structures or viscera, or tumors with less chance of being visualized by transcutaneous ultrasonography (US). To address the clinical existent challenges, a laparoscopic approach may appear to be an appealing alternative for treating large hemangioma [16].

The aim of this study was to evaluate the technical and clinical outcomes of laparoscopic RF ablation for treating a cohort of patients with large subcapsular hepatic hemangiomas.

## Materials and Methods

### Patients

We retrospectively reviewed the data records of consecutive patients with large subcapsular hepatic hemangiomas who had been treated by laparoscopic RF ablation from October 2011 to February 2015. The diagnosis of hepatic hemangioma was based on at least two coincidental radiologic findings on US, contrast-enhanced computed tomography (CT) and magnetic resonance imaging (MRI). Data were collected from the clinical data base of six hospitals in China: Beijing Chaoyang Hospital affiliated with Capital Medical University, Beijing, China; The Fifth Affiliated Hospital of Wenzhou Medical College, Zhejiang, China; Zhanhua People's Hospital, Shandong, China; Chaoyang Central Hospital, Liaoning, China; Affiliated Hospital of Chifeng University, Neimenggu, China; Shanxi Provincial People's Hospital, Shanxi, China.

This study was approved by the ethics committees of each participating hospital, including ethics committees of Beijing Chaoyang Hospital affiliated with Capital Medical University, Beijing, China; The Fifth Affiliated Hospital of Wenzhou Medical College, Zhejiang, China; Zhanhua People's Hospital, Shandong, China; Chaoyang Central Hospital, Liaoning, China; Affiliated Hospital of Chifeng University, Neimenggu, China; Shanxi Provincial People's Hospital, Shanxi, China. Written informed consent was waived considering the retrospective nature of this study and this study was performed in compliance with the Declaration of Helsinki.

From October 2011 to February 2015, a total of 1232 patients with large hepatic hemangiomas (≥ 5 cm) were diagnosed in the above mentioned hospitals. All were initially managed by periodically clinical follow-up. Over time, 212 patients were considered to be the candidates who need further treatments. The indications for treatment are described in our previously published article [12, 14]. Based on our accumulated experience of treating large hepatic hemangiomas with RF ablation [12, 14, 16], we treated these patients with hepatic hemangiomas using RF ablation as the first-line treatment.Of the 212 patients, 121 patients with 124 subcapsular hepatic hemangiomas treated by RF ablation via a laparoscopic approach were included in this study. Inclusion criteria for laparoscopic RF ablation include: (1) hepatic hemangiomas located within 5 mm of the liver capsule; (2) tumors in proximity to abdominal viscera or diaphragm which are contraindicated for RF ablation via percutaneous imaging-guided approaches.

### RF ablation system

RF procedures in this study were performed using Cool-tip ACTC 2025 electrodes and an RF generator (Covidien Healthcare, Dublin, Ireland).

### Laparoscopic RF procedure

After induction of general anesthesia, patients were placed in a supine position. Pneumoperitoneum (CO_2_ at 12 mmHg) was established, and the abdomen was explored with a 30° laparoscope through a 10 mm umbilical port. Another 10 mm subxiphoid port was created at the midline of abdomen. One additional 10 mm right or left lateral subcostal port was placed if needed, depending on the location of the hepatic hemangiomas. Under US guidance, the RF probe was introduced into the peritoneal cavity through the subcostal abdominal wall with laparoscopic visualization and deployed into the tumor. The abdominal wall puncture was positioned as close to the target lesion as possible. The RF process was monitored by intraoperative US. The ablation strategies were described in our previously published article [14].The ablated lesion became hyperechoic because of outgassing from heated tissues. Laparoscopic biopsy of liver lesions before the ablation was not performed to avoid unnecessary bleeding.

For the patients with gallbladder stones or simple hepatic cysts, laparoscopic cholecystectomy (LC) or deroofing of the hepatic cysts was performed during ablation. LC had to be performed beforehand if the lesions were encroaching on the gallbladder fossa.

### Postoperative evaluation

All patients were followed with contrast material-enhanced CT or MRI 1 month after ablation. Complete ablation was defined as no nodular or irregular enhancement adjacent to the ablated zone, as shown on enhanced CT or MRI scans. Incomplete ablation was defined as irregular, peripheral-enhanced foci in the ablated zone. In the case of complete ablation, subsequent CT or MRI examinations were repeated at 6-month intervals. In the case of incomplete ablation, repeat RF ablation was not performed unless the residual tumor had progressed during follow-up at 6-month intervals.

### Study endpoints

Primary endpoints of the study were technical success, safety (complications related to RF ablation), and confirmed complete ablation. Secondary endpoints were alleviation of symptoms, change in the size of the ablation zone, recurrence of the residual tumor, and quality of life. The endpoints of the study were defined at 6 months after the RF ablation treatment.

### Statistical analysis

Values were expressed as means ± SD. Differences in the categorical data were analyzed by use of the χ^2^ test or Fisher’s exact test. Two-tailed *p* values of <0.05 were deemed significant. Statistical analysis was performed using SPSS version 15.0 for Windows (SPSS, Chicago, IL, USA).

## Results

### RF ablation procedure

Of the 121 patients, 40 (33.1%) were male and 81 (66.9%) female. The mean diameter of the 124 hemangiomas was 9.1 ± 3.2 cm (5.0–16.0 cm). One hundred and eighteen patients had a single lesion, 3 patients had 2 lesions (5.2 cm and 11 cm, 5.0 cm and 13 cm, 5.5 cm and 11 cm respectively). The patients’ demographic characteristics and characteristics of the tumors are provided in Tables 1 and 2, respectively.

**Table 1:** Characteristics of 121 patients in the study.

Characteristics	n = 121
**Age (years)**	**49±11 (26–76)**
**Sex (male:female)**	**40:81**
**No. of hemangiomas**	
**Single lesion**	**118(97.5%)**
**Two lesions**	**3 (2.5%)**
**Co-morbidities, N (%)**	
**Gallbladder stones**	**5(4.1%)**
**Type 2 diabetes mellitus**	**7(5.8%)**
**History of open cholecystectomy**	**3(2.5%)**
**Chronic hepatitis B**	**4(3.3%)**
**History of previous liver surgery**	**2(1.7%)**
**Hepatic cysts**	**3(2.5%)**
**Reasons for RF ablation, N (%)**	
**Abdominal pain or discomfort**	**9(7.4%)**
**Enlargement of hemangioma**	**52(43.0%)**
**Abdominal pain and enlargement hemangioma**	**60(49.6%)**

**Table 2:** Characteristics of 124 subcapsular hepatic hemangiomas.

Parameter	(n = 124)
**Size of hemangioma (cm), N (%)**	
**≥ 5 and < 10**	**72 (58.1%)**
**≥ 10**	**52(41.9%)**
**Maximal size of hemangioma, (cm), mean (sd)**	**9.1(3.2)**
**Max**	**16.0**
**Min**	**5.0**
**Adjacent organs, N (%)**	
**Gallbladder**	**8(6.5%)**
**Stomach**	**54(43.6%)**
**Abdominal wall and peritoneum**	**45(36.3%)**
**Colon**	**38(30.7%)**
**Diaphragm**	**35(28.2%)**

**Fig 1:**
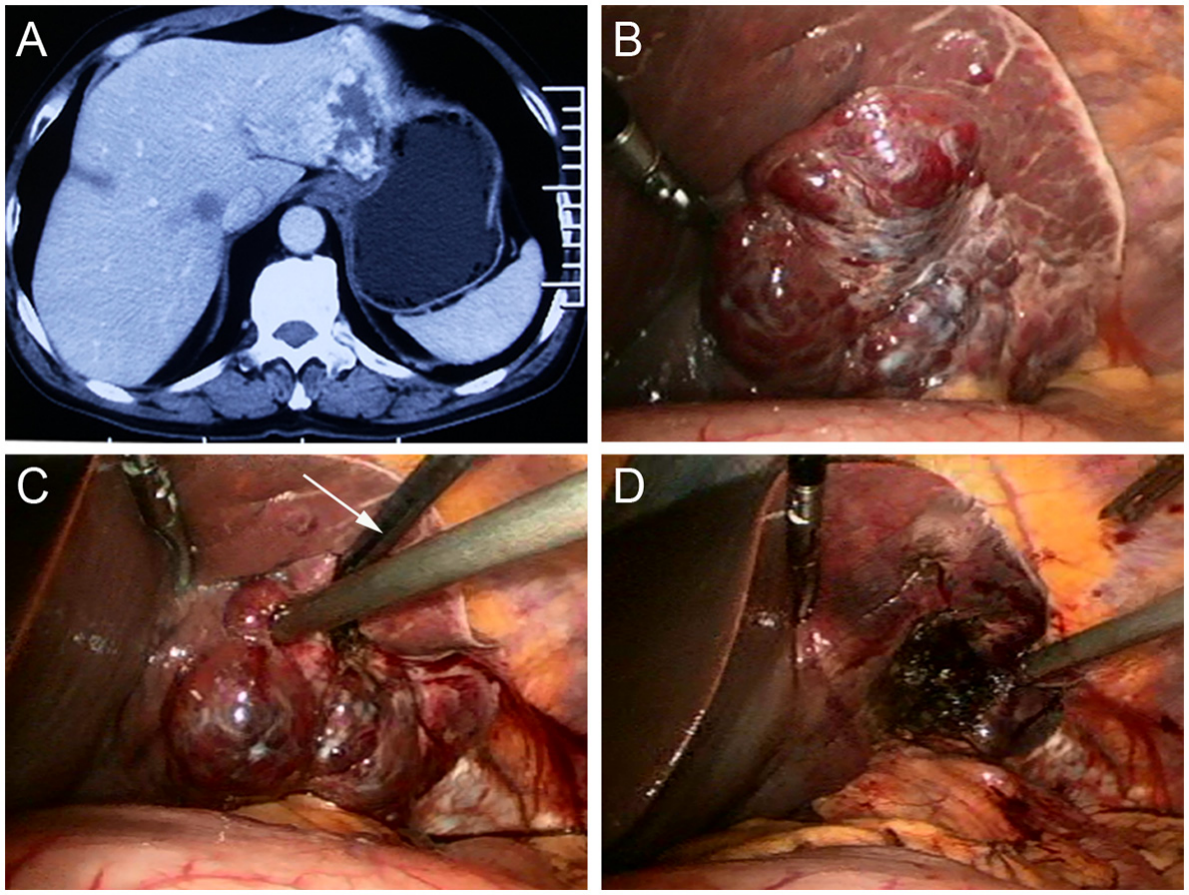
**(A)** A Forty-six-year-old man had an 8.0 cm hemangioma in segment 2 and 3, as seen on an abdominal CT scan. (**B)** On laparoscopic views, the tumor is evident proximity to the stomach. (**C)** The radiofrequency (RF) probe (arrow) was deployed at the edge of tumor when ablation started, where the heat-sink effect is relatively small and bleeding is relatively easy to control. **(D)** The lesion became a depressed mass with hard texture after RF ablation.

Outcome data for the RF ablation treatments are given in Table 3. One hundred and twenty-four subcapsular hemangiomas were treated with laparoscopic RF ablation, and the Pringle maneuver was used in 43 (43/124, 34.7%) lesions. Ablation treatment was conducted according to the pre-defined protocols; there were no technical failures (Fig 1). In the 121 patients, 116 received only a single RF ablation session, but 5 patients (4.1%) with single lesions of 13.0–16.0 cm in diameter, received t, respectively.minating ablationion on the first ablative procedure wo RF ablation sessions; treatment was terminated during the first session in 2 patients when their body temperatures exceeded 39°C and in 3 patients when hemoglobinuria occurred. In these 5 patients, second sessions were conducted under CT-guide percutaneous approach 1 month after the first session (Table 3). LC was performed in 11 patients undergoing laparoscopic RF ablation for prevention of gallbladder injury (n = 6) and gallbladder stones (n = 5). Laparoscopic deroofing was performed in 3 patients during the ablation procedure in patients with hepatic cysts (n = 3). The patients with hemangioma <10 cm had a significantly shorter ablation time (62.2 ± 7.1 min vs. 140.0 ± 18.5 min, *P* < 0.001) and less blood loss (41.1 ± 20.3 vs. 132.5 ± 47.1 mL, *P* < 0.001) compared to the patients with hemangiomas ≥ 10 cm. No patient in the present group required blood transfusion.

**Table 3:** Outcome of laparoscopic radiofrequency (RF) ablation on 124 subcapsular hepatic hemangiomas.

Parameter	(n = 124)
**Technical success rate, N (%)**	**124 (100.0)**
**RF ablation sessions, N (%)**	
**One RF ablation session**	**119 (96.0)**
**Two RF ablation sessions**	**5 (4.0)**
**Complete ablation, N (%)**	**118 (95.2)**
**No. of punctures per lesion, mean (sd)**	**5.5 (1.1)**
**Time of ablation per lesion, min, mean (sd)**	**98.3 (40.5)**
**Diameter of ablated zone 1 month after ablation, cm, mean (sd)**	**7.0 (1.5)**
**Diameter of ablated zone 6 months after ablation, cm, mean (sd)**	**5.4 (1.4)**

### Safety of RF ablation: ablation-related complications

As illustrated in Table 4, there were 66 complications related to the ablation in 26 patients, including 5 of 69 (7.3%) patients with hemangioma <10 cm and 21 of 52 (40.4%) patients with hemangiomas ≥ 10 cm (*P* < 0.001).

**Table 4:** Complications of laparoscopic radiofrequency (RF) ablation for 121 patients.

Complications	Size ≥ 5 cm and < 10 cm (n = 69)	Size ≥ 10 cm (n = 52)	*P* value
**Total no. of patients with complication, N (%)**	**5(7.3)**	**21(40.4)**	**<0.001**
**Incidence of complication, N (%)**			
**Hemoglobinuria**	**4(5.8)**	**21(40.4)**	**<0.001**
**Fever** *	**1(1.5)**	**13(25.0)**	**<0.001**
**Hemolytic jaundice** ‡	**0(0.0)**	**8(15.4)**	**0.001**
**Anemia** #	**0(0.0)**	**2(3.9)**	**0.183**
**Elevated serum transaminase** **	**3(4.4)**	**12(23.1)**	**0.004**
**Skin burns**	**0(0.0)**	**0(0.0)**	
**Transient renal damage** †	**0(0.0)**	**0(0.0)**	
**Visceral damage**	**0(0.0)**	**0(0.0)**	

* Fever ≥ 38°C.

^‡^ Total bilirubin > 34.2 μmol/L.

^#^ hemoglobin < 100 g/L.

** Serum transaminase > 80 U/L.

^†^ Creatinine > 200 μmol/L.

Among individual complications, patients with hemangiomas < 10 cm had significantly less hemoglobinuria than did patients hemangiomas ≥10 cm (5.8% vs. 40.4%, *P* < 0.001); there was a trend also for less hemolytic hyperbilirubinemia (total bilirubin > 34.2 μmol/L) (*P* = 0.001), fever (≥ 38°C) (*P* < 0.001), elevated serum transaminase(including alanine transaminase and aspartate transaminase) (> 80 U/L) (*P* = 0.004) and anemia (hemoglobin <100 g/L) (*P* = 0.183) in the patients with hemangiomas <10 cm.

No injuries to abdominal viscera occurred in all the 121 patients. According to the Dindo–Clavien classification [17], all the complications were minor in 26 patients (Grade I).

Twenty-five patients developed hemoglobinuria and were managed with adequate hydration to maintain at least 100 mL per hour of urine output. All hemoglobinuria subsided within 72 hours. Of the 25 patients who had hemoglobinuria, 8 had hemolytic jaundice (total bilirubin >34.2 mmol/L) and 2 patients developed anemia (hemoglobin <100 g/L). All these abnormalities resolved spontaneously within 1 month and did not require major pharmacologic intervention or blood transfusion. Fourteen patients experienced moderate fevers from 38°C to 39°C that persisted for up to 3 to 6 days; all resolved spontaneously and did not require treatment with antibiotics. Increased serum transaminase (>80 U/L), including alanine transaminase and aspartate transaminase, developed in 15 patients, which resolved spontaneously within 2 weeks after ablation.

The hospital stay of the 121 patients was 2–13 (4.1±2.6) days. The hospital stay of patients with hemangiomas < 10 cm was significantly shorter than the patients hemangiomas ≥10 cm (3.2±1.5 vs. 5.3±3.2 days, *P*<0.01).

### Efficacy of RF ablation

Out of 124 hepatic hemangiomas, 118 (95.2%) were completely ablated, including 70 of 72 (97.2%) lesions < 10 cm and 48 of 52 (92.3%) lesions ≥ 10 cm (*P* = 0.236). Five large hemangiomas (≥ 13 cm) were completely ablated after 2 RF ablation sessions. Six hemangiomas were incompletely ablated, showing subtle enhancement on the peripheral rim of the ablated tumors on follow-up CT or MRI.

### Follow-up results

After RF ablation, there was no perioperative mortality or delayed complications, such as local tumor progression, destructive biliary damages, or liver abscess. The mean diameter of ablation zone was decreased to 7.0 ± 1.5 cm one month after ablation and further decreased to 5.4 ± 1.4 cm in last follow-up. The 6 residual lesions also shrunk but not obviously during the follow-up period and necessitated no further treatment. Of the 69 patients with obvious symptoms related to hemangioma, 63 patients had a complete disappearance of their symptoms and 6 ameliorated without any therapy after ablation.

At the 6-month follow-up, no patient had developed new symptoms attributable to the hemangiomas. The subjective health status and quality of life were rated as good to excellent in 100% of patients at follow-up. After RF ablation of the hemangiomas, all patients were able to perform full-time or part-time work.

## Discussion

Our study evaluated the technical and clinical outcomes of laparoscopic RF ablation as an alternative treatment for a cohort of patients with large subcapsular hepatic hemangiomas. The results of our study show that laparoscopic RF ablation is a feasible, safe and effective technique for treating large subcapsular hepatic hemangiomas with absence of severe complications, such as accidental visceral injuries.

RF ablation is an effective treatment option for early hepatocellular carcinoma with comparable results to surgical resection in selected patients [18, 19]. The percutaneous approach to RF ablation is relatively simple to perform and has the advantage of not requiring a surgical procedure. However, the tumor adjacent to the gallbladder or other viscera precludes a percutaneous approach because of the potential thermal injury and the consequent risk of perforation. The incidence of visceral damage is varying from 0.5% to 0.7% in the literature [20]. Damages to the colon, stomach, gallbladder, kidney, diaphragm, abdominal wall and small intestine have been described [20]. Although the incidence of visceral damage is rare, most of the complications were fatal even if the patients were early diagnosed and adequately treated. Risk factors are percutaneous approach, subcapsular tumors, previous abdominal surgery and chronic cholecystitis as the patient may have adhesions between the liver and the bowel. Livraghi et al [21] suggest some issues in these patients: they should be treated by the open or laparoscopic approach for direct visualization of the organs, assuring they are substantially separated.

It is more difficult and risky to use percutaneous RF ablation for treating subcapsular hepatic hemangiomas than subcapsular hepatic malignant tumors, because hemangiomas to be treated are usually larger than malignant tumors and have wider contact area with the viscera. With the help of laparoscopic approach, this difficulty and the risk can be solved to the largest extent. The laparoscopic technique offers a direct vision of the entire procedure, allowing not only the isolation of the tumour from the abdominal viscera, but also performing simultaneous procedures such as a cholecystectomy, deroofing of hepatic cyst and liver resection. Intraoperative US were used routinely in conjunction with the laparoscopic approach to increase the ability to guide the RF electrode placement and evaluate the efficacy of ablation [22–24]. Furthermore, capnopneumoperitoneum adds a beneficial physiopathological effect to RF ablation as it decreases the portal inflow by 40% allowing a better thermal conduction, which contributes to a better ablation efficacy [22].

Laparoscopic RF ablation has been shown effective and reasonably safe in the treatment of subcapsular hemangiomas of 5 to 9.9 cm in size by two independent study groups [10, 13]. However, the two studies [10, 13] only recruited patients with subcapsular hemangiomas smaller than 10 cm for laparoscopic RF ablation, because authors were afraid of high complication rates and treatment failure for hepatic hemangiomas larger than 10 cm in size [11]. The study shows that complete and sustained resolution of subcapsular hepatic hemangiomas was attained undergoing laparoscopic ablation, even in the patients with hepatic hemangiomas ≥ 10 cm. A similarly high frequency of complete ablation (95.2% vs. 92.3%) of both tumors < 10 cm and ≥ 10 cm was attained. Although a higher rate of complications was seen in the treatment of hemangiomas ≥ 10 cm, all of which were trivial. No severe morbidities or mortality was observed in all the 121 patients. We instituted two measures to treat hepatic hemangiomas ≥ 10 cm in the hope of having few complications, while still maintaining a successful rate of ablation: (1) Cool-tip cluster electrodes were used because of their efficiency and more concentrated release of heat, which are features that can reduce the occurrence of hyperthermia and hemoglobulinuria. (2) During the procedure, if the patient’s body temperature exceeded 39°C or hemoglobinuria occurred, they stopped the procedure and scheduled a repeat session [12, 14]. As a result, 5 patients (4.1%) with single lesions of 13.0–16.0 cm in diameter received t, respectively.minating ablationion on the first ablative procedure wo RF ablation sessions. The ablation was terminated during the first session in 2 patients when their body temperatures exceeded 39°C and in 3 patients when hemoglobinuria occurred. The remnant tumor tissues of the 5 hemangiomas were situated the deep locations of liver after the laparoscopic ablative approach. We therefore suggest that the laparoscopic approach of RF ablation should be the first-line treatment for subcapsular hepatic hemangiomas. If a second ablation session is needed, the repeat RF ablation would be performed percutaneously.

To the best of our knowledge, our study involved the largest cohort of patients with large hepatic hemangiomas treated in with laparoscopic RF ablation [10, 13]. A recent report by Zhang et al [13] evaluated the outcomes of laparoscopic RF ablation (32 patients) compared with conventional open resection (34 patients) for hepatic hemangiomas (4 cm ≤ diameter < 10 cm). They found that laparoscopic ablation significantly shortened the operative time and less blood loss compared with open resection. Furthermore, patients treated by laparoscopic ablation experienced significantly less pain and required less administration of analgesia. Patients with laparoscopic RF ablation need significantly shorter length of hospital stay and lower hospital cost. The overall morbidity rate was similar between the two groups of patients. Another study [25] retrospectively analyzed 86 patients with liver hemangioma larger than 10 cm treated by enucleation (46 patients) or liver resection (40 patients). Results showed that mean blood loss of 86 patients was 526.1± 468.8 mL (range, 100–3000) and 13 (15.1%) patients received blood transfusion. Twenty-eight (32.6%) patients had complications, including pleural effusion (22 patients), diaphragmatic injury (1 patient), hemorrhage (3 patient), pneumonia (1 patient), and bile leak (1 patient). The reported median postsurgical hospital stay was 12 days. In our study, laparoscopic RF ablation for treating large hepatic hemangiomas (≥ 10 cm) experienced less blood loss and shorter hospital stay than the cohort of patients treated by surgical resection [25]. Laparoscopic RF ablation is much less invasive, much safer and cost-effective than surgical resection for managing large hepatic hemangiomas.

The major limitations of our study include its retrospective nature, the lack of control group, the short follow-up period and the relatively small number of patients evaluated. Feasibility for RF ablation is largely dependent on the operator’s technique, experience, and the instrumental equipment of the center. The present patients were managed based on the treating surgeon’s perspective as well as by a team of surgeons, making the results less applicable to nonsurgical clinics. Nevertheless, our data may be helpful for clinicians who treat subcapsular hepatic hemangiomas with RF ablation and may also be useful as a basis for the design of future trials. Again, more long-term outcomes and prospective randomized control trials are needed to define the role of laparoscopic RF ablation in the treatment of subcapsular hepatic hemangiomas, especially in comparison to surgical resection.

In conclusion, laparoscopic RF ablation therapy is a safe, feasible and effective procedure for subcapsular hepatic hemangiomas, even in the hepatic hemangiomas ≥ 10 cm. Its use avoids thermal injury to the abdominal viscera.

## Supporting Information

S1 Fig (A) A Forty-six-year-old man had an 8.0 cm hemangioma in segment 2 and 3, as seen on an abdominal CT scan. (B) On laparoscopic views, the tumor is evident proximity to the stomach. (C) The radiofrequency (RF) probe (arrow) was deployed at the edge of tumor when ablation started, where the heat-sink effect is relatively small and bleeding is relatively easy to control. (D) The lesion became a depressed mass with hard texture after RF ablation.
(TIF)

S1 Table Characteristics of 121 patients in the study. (DOC)

S2 Table Characteristics of 124 subcapsular hepatic hemangiomas. (DOC)

S3 Table Outcome of laparoscopic radiofrequency (RF) ablation on 124 subcapsular hepatic hemangiomas. (DOC)

S4 Table Complications of laparoscopic radiofrequency (RF) ablation for 121 patients. (DOC)
