# Supplementary figures and images for: Laparoscopic Radiofrequency Ablation for Large Subcapsular Hepatic Hemangiomas: Technical and Clinical Outcomes
Source: PLoS One. 2016 Feb 22;11(2):e0149755. doi: 10.1371/journal.pone.0149755 (PMC4765839; doi:10.1371/journal.pone.0149755)

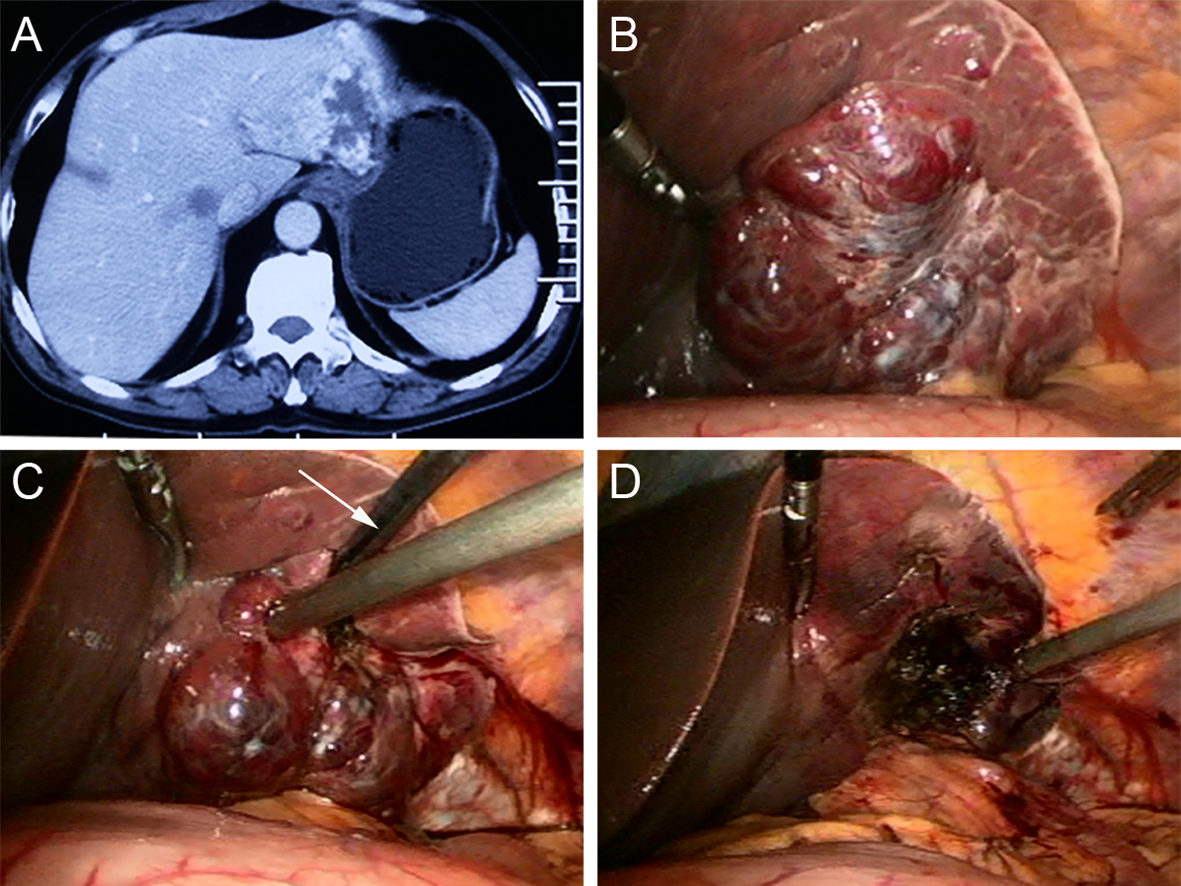

Supplement: S1 Fig — (A) A Forty-six-year-old man had an 8.0 cm hemangioma in segment 2 and 3, as seen on an abdominal CT scan. (B) On laparoscopic views, the tumor is evident proximity to the stomach. (C) The radiofrequency (RF) probe (arrow) was deployed at the edge of tumor when ablation started, where the heat-sink effect is relatively small and bleeding is relatively easy to control. (D) The lesion became a depressed mass with hard texture after RF ablation. (TIF) [file pone.0149755.s001.tif]
