# Supplementary material for: Laparoscopic Radiofrequency Ablation for Large Subcapsular Hepatic Hemangiomas: Technical and Clinical Outcomes
Source: PLoS One. 2016 Feb 22;11(2):e0149755. doi: 10.1371/journal.pone.0149755 (PMC4765839; doi:10.1371/journal.pone.0149755)
Supplement: S1 Table — (DOC) [file pone.0149755.s002.doc]

**Table 1. Characteristics of 121 patients in the study.**

| **Characteristics** | **n= 121** |
| --- | --- |
| **Age (years)** | **49±11 (26-76)** |
| **Sex (male:female)** | **40:81** |
| **No. of hemangiomas** |  |
| **Single lesion** | **118(97.5%)** |
| **Two lesions** | **3 (2.5%)** |
| **Co-morbidities, N (%)** |  |
| **Gallbladder stones** | **5(4.1%)** |
| **Type 2 diabetes mellitus** | **7(5.8%)** |
| **History of open cholecystectomy** | **3(2.5%)** |
| **Chronic hepatitis B** | **4(3.3%)** |
| **History of previous liver surgery** | **2(1.7%)** |
| **Hepatic cysts** | **3(2.5%)** |
| **Reasons for RF ablation, N (%)** |  |
| **Abdominal pain or discomfort** | **9(7.4%)** |
| **Enlargement of hemangioma** | **52(43.0%)** |
| **Abdominal pain and enlargement hemangioma** | **60(49.6%)** |
