# Supplementary material for: Laparoscopic Radiofrequency Ablation for Large Subcapsular Hepatic Hemangiomas: Technical and Clinical Outcomes
Source: PLoS One. 2016 Feb 22;11(2):e0149755. doi: 10.1371/journal.pone.0149755 (PMC4765839; doi:10.1371/journal.pone.0149755)
Supplement: S2 Table — (DOC) [file pone.0149755.s003.doc]

**Table 2. Characteristics of 124 subcapsular hepatic hemangiomas.**

| **Parameter** | **(n= 124)** |
| --- | --- |
| **Size of hemangioma (cm), N (%)** |  |
| **≥ 5 and < 10** | **72 (58.1%)** |
| **≥ 10** | **52(41.9%)** |
| **Maximal size of hemangioma, (cm), mean (sd)** | **9.1(3.2)** |
| **Max** | **16.0** |
| **Min** | **5.0** |
| **Adjacent organs, N (%)** |  |
| **Gallbladder** | **8(6.5%)** |
| **Stomach** | **54(43.6%)** |
| **Abdominal wall and peritoneum** | **45(36.3%)** |
| **Colon** | **38(30.7%)** |
| **Diaphragm** | **35(28.2%)** |
