# Supplementary material for: Laparoscopic Radiofrequency Ablation for Large Subcapsular Hepatic Hemangiomas: Technical and Clinical Outcomes
Source: PLoS One. 2016 Feb 22;11(2):e0149755. doi: 10.1371/journal.pone.0149755 (PMC4765839; doi:10.1371/journal.pone.0149755)
Supplement: S3 Table — (DOC) [file pone.0149755.s004.doc]

**Table 3. Outcome of laparoscopic radiofrequency (RF) ablation on 124 subcapsular hepatic hemangiomas.**

| **Parameter** | **(n= 124)** |
| --- | --- |
| **Technical success rate, N (%)** | **124 (100.0)** |
| **RF ablation sessions, N (%)** |  |
| **One RF ablation session** | **119 (96.0)** |
| **Two RF ablation sessions** | **5 (4.0)** |
| **Complete ablation, N (%)** | **118 (95.2)** |
| **No. of punctures per lesion, mean (sd)** | **5.5 (1.1)** |
| **Time of ablation per lesion, min, mean (sd)** | **98.3 (40.5)** |
| **Diameter of ablated zone 1 month after ablation, cm, mean (sd)** | **7.0 (1.5)** |
| **Diameter of ablated zone 6 months after ablation, cm, mean (sd)** | **5.4 (1.4)** |
