# Supplementary material for: Laparoscopic Radiofrequency Ablation for Large Subcapsular Hepatic Hemangiomas: Technical and Clinical Outcomes
Source: PLoS One. 2016 Feb 22;11(2):e0149755. doi: 10.1371/journal.pone.0149755 (PMC4765839; doi:10.1371/journal.pone.0149755)
Supplement: S4 Table — (DOC) [file pone.0149755.s005.doc]

**Table 4. Complications of laparoscopic radiofrequency (RF) ablation for 121 patients.**

| **Complications** | **Size ≥ 5 cm and < 10 cm (n=69)** | **Size ≥ 10 cm (n= 52)** | ***P* value** |
| --- | --- | --- | --- |
| **Total no. of patients with complication, N (%)** | **5(7.3)** | **21(40.4)** | **<0.001** |
| **Incidence of complication, N (%)** |  |  |  |
| **Hemoglobinuria** | **4(5.8)** | **21(40.4)** | **<0.001** |
| **Fever *** | **1(1.5)** | **13(25.0)** | **<0.001** |
| **Hemolytic jaundice ‡** | **0(0.0)** | **8(15.4)** | **0.001** |
| **Anemia #** | **0(0.0)** | **2(3.9)** | **0.183** |
| **Elevated serum transaminase **** | **3(4.4)** | **12(23.1)** | **0.004** |
| **Skin burns** | **0(0.0)** | **0(0.0)** |  |
| **Transient renal damage †** | **0(0.0)** | **0(0.0)** |  |
| **Visceral damage** | **0(0.0)** | **0(0.0)** |  |

* Fever ≥ 38°C. ‡ Total bilirubin > 34.2 μmol/L. # hemoglobin < 100 g/L. ** Serum transaminase > 80 U/L. † Creatinine > 200 μmol/L.
